# Genetic Diversity and Molecular Evolution of *Plum bark necrosis stem pitting-associated virus* from China

**DOI:** 10.1371/journal.pone.0105443

**Published:** 2014-08-21

**Authors:** Linning Qu, Hongguang Cui, Guanwei Wu, Jufang Zhou, Jiaming Su, Guoping Wang, Ni Hong

**Affiliations:** 1 College of Plant Science and Technology, Huazhong Agricultural University, Wuhan, Hubei, China; 2 National Key Laboratory of Agromicrobiology, Huazhong Agricultural University, Wuhan, Hubei, China; 3 Yantai Agricultural Science and Technology Institute, Yantai Academy of Agricultural Science, Yantai, Shandong, China; Institute of Infectious Disease and Molecular Medicine, South Africa

## Abstract

*Plum bark necrosis stem pitting-associated virus* (PBNSPaV), a member of the genus *Ampelovirus* in the family *Closteroviridae*, infects different *Prunus* species and has a worldwide distribution. Yet the population structure and genetic diversity of the virus is still unclear. In this study, sequence analyses of a partial heat shock protein 70 homolog (HSP70h) gene and coat protein (CP) gene of PBNSPaV isolates from seven *Prunus* species grown in China revealed a highly divergent Chinese PBNSPaV population, sharing nucleotide similarities of 73.1–100% with HSP70h gene, and 83.9–98.6% with CP gene. Phylogenetic analysis of HSP70h and CP sequences revealed segregation of global PBNSPaV isolates into four phylo-groups (I–IV), of which two newly identified groups, II and IV, solely comprised Chinese isolates. Complete genome sequences of three PBNSPaV isolates, Pch-WH-1 and Pch-GS-3 from peaches, and Plm-WH-3 from a plum tree, were determined. The three isolates showed overall nucleotide identities of 90.0% (Pch-GS-3) and 96.4% (Pch-WH-1) with the type isolate PL186, and the lowest identity of 70.2–71.2% with isolate Nanjing. For the first time, to the best of our knowledge, we report evidence of significant recombination in the HSP70h gene of PBNSPaV variant Pch2 by using five programs implemented in RDP3; in addition, five codon positions in its CP gene (3, 8, 44, 57, and 88) were identified that appeared to be under positive selection. Collectively, these results indicate a divergent Chinese PBNSPaV population. In addition, our findings provide a foundation for elucidating the epidemiological characteristics of virus population.

## Introduction

*Plum bark necrosis stem pitting-associated virus* (PBNSPaV) is a member of the genus *Ampelovirus* in the family *Closteroviridae* [1]. The stem-pitting disease of sweet cherry trees was described in the 1960s in North America [2], [3]. Subsequently, the disease has been observed in many cultivated and ornamental *Prunus* species, including Japanese plum (*Prunus salicina*), apricot (*P. armeniaca*), peach (*P. persica*), cherry (*P. avium*), and almond (*P. dulcis*) [4]–[9]. To date, the disease has been reported in Italy [4], Morocco [10], Serbia [11], [12], Jordan [13], Egypt [14], Turkey [15], France [16], and China [17]. Some diseased trees show decline, gummosis, flattening of scaffold branches, severe necrosis of bark tissues, and necrotic pitting on the woody cylinders [18], [19]. The plum bark necrosis-stem pitting disease was first confirmed to be graft-transmissible on a Japanese plum cv. Black Beaut grown in the United States [20]. High-molecular-weight double-stranded RNAs (dsRNA) were first recovered from symptomatic cherry trees in California [21]. The first successful amplification of cDNA fragments, using HSP70h (a homolog of the heat shock protein 70 gene)-degenerate primers for viruses in the family *Closteroviridae*, identified the virus as a closterovirus, and it was named *Plum bark necrosis stem pitting-associated virus* (PBNSPaV) [7]. The association of the virus with the PBNSP disease of stone fruit trees was further confirmed by using degenerate primers, and primers designed within the fragment amplified by the degenerate primers [4], [22]. The first complete genome sequence of a PBNSPaV isolate, PL186, was determined by Al Rwahnih *et* *al.* [23]. Its plus and single-stranded RNA genome of 14,214 nucleotides (nts) long consists of seven open reading frames (ORFs), and two untranslated regions (UTRs) at the 5′ and 3′ termini. ORFs 1a and 1b encode a large polyprotein with a molecular mass (Mr) of 259.6 kDa, containing conserved domains characteristic of a papain-like protease, a methyltransferase, and a helicase (ORF1a), and a 64.1-kDa RNA-dependent RNA polymerase (RdRp) (ORF1b), respectively. ORF2 and ORF4 encode a 6.3-kDa hydrophobic protein and a 61.6-kDa protein of unknown function, respectively. ORF3 encodes a 57.4-kDa heat shock protein homolog (HSP70h). ORF5 and ORF6 encode a 35.9-kDa capsid protein (CP) and a 25.2-kDa minor capsid protein (CPm), respectively. The molecular characterization of the virus supported its classification in the genus *Ampelovirus* [23]. Recently, the complete genome sequences of four PBNSPaV isolates have been determined by pyrosequencing, and genome sequence comparison showed that the PBNSPaV population is diverse; a PBNSPaV isolate named ‘Nanjing,’ from a Chinese peach sample, was found to be highly divergent, showing only 74.4–76.9% identity to other PBNSPaV isolates [16].

China is an important country for fruit production, and stone fruit trees and ornamental trees in the genus *Prunus* are grown in almost all regions of China. However, the incidence and distribution of PBNSPaV in the various *Prunus* species grown in China is unknown. Recently, during field investigations for the incidence of viral diseases in stone fruit trees in China, some cultivated and ornamental *Prunus* species were found to show trunk gummosis, stem pitting or grooving and some plum trees were found to have died from the disease. The presence and divergence of PBNSPaV isolates in China was first confirmed by reverse transcription (RT)-PCR and sequencing of a 590-nt fragment of HSP70h gene [17]. The origins and evolutionary status of PBNSPaV isolates are poorly understood. Given the apparent effect of the disease associated with PBNSPaV on fruit production, it is imperative to better understand the genetic variation and population structure of PBNSPaV. In this study, PBNSPaV isolates from different *Prunus* species grown in China were characterized by sequencing their CP and HSP70h genes; the complete genomes of three representative isolates were also sequenced. The objectives of this study were (i) to determine the incidence of PBNSPaV in *Prunus* trees grown in China, and (ii) to characterize the genetic variability of the PBNSPaV isolates obtained from different *Prunus* species and from various regions of China. This study provides useful information for enhanced understanding of molecular evolution within the global PBNSPaV population, which will be helpful for epidemiological investigations and for developing more efficient molecular detection methods for the diagnosis of the viral disease.

## Results

### RT-PCR analysis shows wide PBNSPaV infections in stone fruit trees

**Figure 1:**
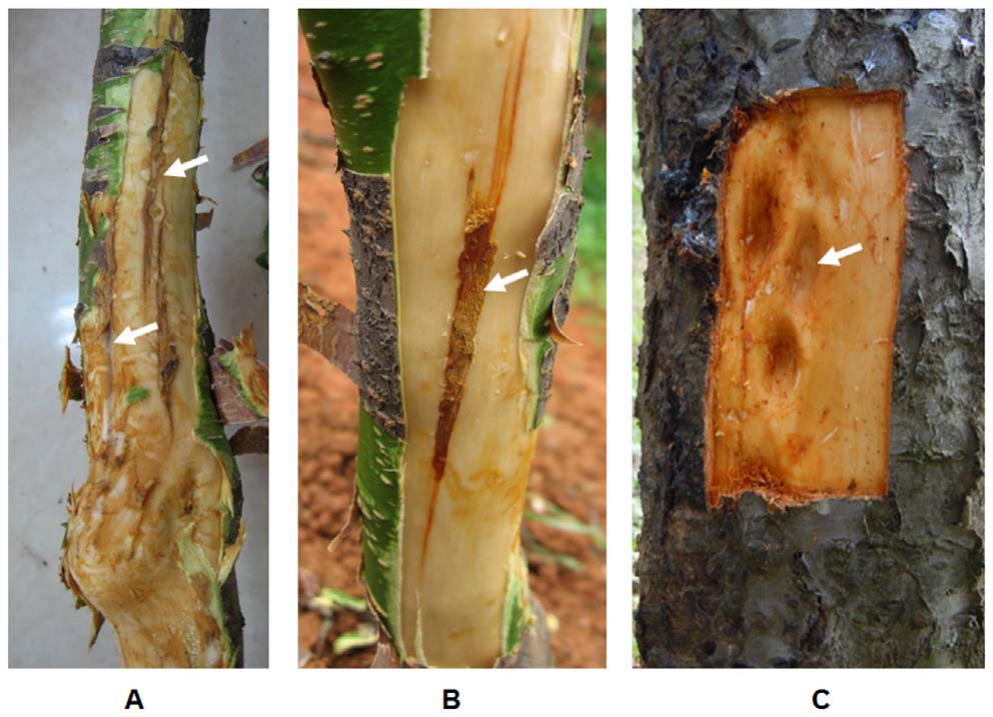
Stem grooving (A) and brown necrotic lines (B) on the trunks of plum trees, and stem pitting on the trunk of a plum tree.

RT-PCR using the primer pair PBN-HSP-P1/PBN-HSP-P2 showed that of the 256 samples analyzed, 52 samples (20.3%), including 25 from peach, 5 from flowering peach, 1 from nectarine, 10 from plum, 1 from apricot, 7 from cherry, and 3 from flowering cherry, were positive for PBNSPaV. In peach, flowering peach, and plum trees, PBNSPaV was usually associated with symptoms such as stem pitting and bark cracks on the trunks (Table 1, Fig. 1), whereas other trees were symptomless or showed only dark-colored gummosis.

**Table 1:** Isolates of PBNSPaV collected from China and sequenced for their HSP70 and CP genes.

Isolate	Host	Origin	Symptom^a^	RT-PCR		
				195F/R	HSP-F/R	CP-F/R
Pch-WH-1	Peach	Wuhan	TG\CR\NC\SP	+	+	+
Pch-WH-2	Peach	Wuhan	TG\CR\NC		+	+
Pch-WH-3	Peach	Wuhan	TG\CR\NC		+	+
Pch1	Peach	Wuhan	TG\CR\NC\SP	+	+	+
Pch2	Peach	Wuhan	TG\CR\NC\SP	+	+	+
Pch3	Peach	Wuhan	TG\CR\NC\SP	+	+	+
Pch4	Peach	Wuhan	TG\CR\NC\SP	+	+	
Pch5	Peach	Wuhan	TG\CR\NC\SP	+	+	+
Pch6	Peach	Wuhan	TG\CR\NC\SP	+	+	
Pch7	Peach	Wuhan	NA	+	+	+
Pch-WH-10	Peach	Wuhan	TG\CR\NC	+		+
Pch-WH-11	Peach	Wuhan	TG\CR\NC\SP	+		+
Pch-WH-12	Peach	Wuhan	TG\CR\NC	+	+	+
Pch-WH-13	Peach	Wuhan	TG\CR\SP	+	+	+
Pch-WH-14	Peach	Wuhan	TG\CR	+		+
Pch-WH-15	Peach	Wuhan	TG\CR	+		+
Pch-XN-1	Peach	Xianning	NA	+	+	+
Pch-XN-3	Peach	Xianning	NA	+	+	+
Pch-XN-4	Peach	Xianning	NA	+	+	
Pch-XN-5	Peach	Xianning	NA	+	+	+
Pch-XN-6	Peach	Xianning	NA	+	+	+
Pch-XN-7	Peach	Xianning	NA	+	+	
Pch-XN-8	Peach	Xianning	NA	+		+
Pch-JX-1	Peach	Jiangxi	NA		+	
Fpch-WH-1	Peach	Wuhan	TG\CR		+	
Fpch-WH-2	Peach	Wuhan	TG\CR\NC		+	
Fpch-WH-3	Peach	Wuhan	TG\CR\NC		+	
Fpch-WH-7	Peach	Wuhan	TG\CR\NC			+
Fpch8	Peach	Wuhan	TG\CR\NC\SP	+	+	
Fpch9	Peach	Wuhan	TG\CR\NC\SP	+	+	+
Fpch10	Peach	Wuhan	TG\CR\NC\SP	+	+	
Fpch11	Peach	Wuhan	TG\CR\NC\SP	+	+	
Fch-WH-3	flowering cherry	Wuhan	TG\CR\NC	+		+
Fchr16	flowering cherry	Wuhan	TG\CR\NC\SP	+	+	+
Fchr17	flowering cherry	Wuhan	TG\SP		+	
Plm-WH-1	plum	Wuhan	TG\CR\NC\SP	+	+	
Plm-WH-2	plum	Wuhan	NA	+	+	
Plm-WH-3	plum	Wuhan	TG\CR\NC	+	+	+
Plm-WH-4	plum	Wuhan	TG\CR\NC\SP		+	
Plm12	plum	Wuhan	TG\CR\NC\SP	+	+	
Plm13	plum	Wuhan	NA	+	+	+
Plm-XN-1	plum	Xianning	NA	+	+	+
Plm-XN-2	plum	Xianning	NA		+	
Plm-XN-3	plum	Xianning	NA	+	+	+
Fplm14	flowering plum	Wuhan	TG\SP	+	+	+
Chr15	cherry	Yantai	NA		+	
Chr19	cherry	Yantai	NA	+		+
Chr20	cherry	Yantai	NA	+		+
Ch-YT-1	cherry	Yantai	NA	+		+
Ch-YT-2	cherry	Yantai	NA		+	
Ch-YT-4	cherry	Yantai	NA	+	+	+
Nec18	nectarine	Wuhan	TG\CR\NC	+	+	+
Apr-SX-1	apricot	Shanxi	NA	+	+	+
Apr-YT-1	apricot	Yantai	NA	+	+	+
Pch-LN-2	Peach	Liaoning	NA	+	+	+
Pch-GS-1	Peach	Gansu	NA		+	
Pch-GS-2	Peach	Gansu	NA		+	
Pch-GS-3	Peach	Gansu	NA		+	+
Pch-GS-4	Peach	Gansu	NA		+	+
Total					49	38

a TG: trunk gummosis, CR: cracking, NC: necrosis, SP: stem pitting, NA: not observed.

### Phylogenetic analysis for HSP70h and CP sequences of PBNSPaV isolates reveals existence of divergent variants

The sequences of 87 HSP70h clones from 31 isolates and 61 CP clones from 38 isolates were determined (Table 1). The product sizes for the HSP70h and CP genes were 590 bp and 1041 bp (including 978 bp of the complete CP gene and 48∼63 bp of the intergenic region preceding the CP gene), respectively. The GenBank accession numbers for the determined sequences are KJ792814 to KJ792828 for the CP gene, KJ792829 to KJ792851 for the HSP70h gene. Sequence comparison showed that PBNSPaV isolates from China were highly divergent and the HSP70h sequences (including the 18 sequences reported previously) shared 73.1–100% nt and 86.8–100% amino acid (aa) similarities, respectively. The CP gene sequences of the 38 Chinese isolates shared 74.3–98.6% nt and 87.0%–99.1% aa similarities, respectively. Intra-isolate sequence diversity was found in isolates Pch-GS-3 and Pch-XN-3. The level of nucleotide sequence identity ranged from 73.6–99.3% among distinct HSP70h variants of Pch-GS-3, and from 76.7–86.2% among distinct CP variants of Pch-XN-3.

**Figure 2:**
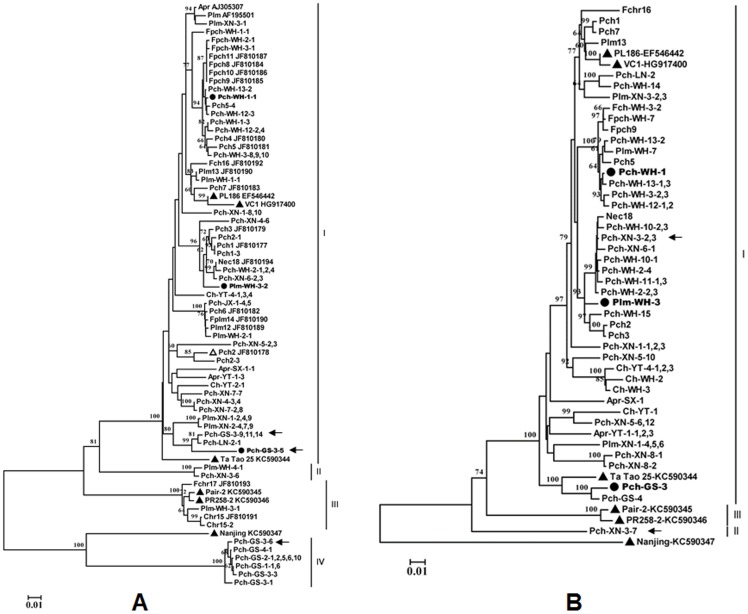
Phylogenetic analysis of isolates of Plum bark necrosis stem pitting-associated virus (PBNSPaV) based on the nucleotide sequences of their partial HSP70h gene (590 nt) (A) and complete coat protein gene (B). The CP sequences of 38 isolates obtained in this study, the partial HSP70 sequences of 41 isolates obtained in this study, and 18 Chinese isolates previously reported were included in the analysis. The six isolates for which the complete genome sequence are available from GenBank are shown with “▴”. The three isolates for which the complete genomes were sequenced in the present study are indicated by “•”. Sequences previously reported by our group and the corresponding sequences available from GenBank are identified by their GenBank accession number following their isolate names. The divergent HSP70h variants within isolate Pch-GS-3 and the CP variants within isolate Pch-XN-3 are indicated by black arrows. The trees were constructed using the neighbor-joining method. The numbers at the nodes indicate bootstrap support (1,000 replicates). Values below 60% are not shown. The scale bar represents 0.01 substitutions per site.

Phylogenetic analysis of the HSP70h nucleotide sequences from Chinese isolates determined in the present study, those previously reported by our group, and eight isolates available in GenBank (Table S1 in File S1) using the neighbor-joining method revealed four major groups (designated as groups I–IV) with strong (100%) bootstrap support values (Fig. 2A). Among those groups, group I and group II are separated by a bootstrap value of 81%, and group IV is separated from the isolate Nanjing KC5990347 by a bootstrap value of 100%. Group I was the largest and contained isolates or variants from all species sampled (81.3% of all obtained sequences) and four sequences (AJ305307, AF159901, EF546442, and KC590344) referred from GenBank. Group II contained only two Chinese isolates from a plum and a peach samples. Group III contained six sequences, including one sequence (Plm-WH-3-1) determined in the present study, three sequences (Fch-WH-2-1, Ch-WH-1-1, Ch-WH-1-2) previously reported by our group (Cui *et* *al.*, 2011), and two sequences (KC590345 and KC590346) of isolates, Pair-2 and PR258-2, from France, recently reported by Marais *et* *al.* [16]. Therefore, group III appears to correspond to Group 2 identified by Marais *et* *al.* [16], based on nucleotide sequences of PBNSPa-3f/2r (HSP70h). Four isolates from peach grown in Gansu province formed a separate group, group IV, which was most closely related to isolate Nanjing (KC590347), sharing 81.6–82.4% nt and 92.3–92.9% aa similarities.

The phylogenetic tree inferred from the nucleotide sequences of the complete CP gene sequences showed a similar topology to the tree inferred from the HSP70h sequences (Fig. 2B); most sequences clustered in a large group with sequence EF546442, obtained from the plum PBNSPaV type isolate PL186. However, no group corresponding to the HSP70h group IV was identified in the analysis of the viral CP sequences.

Sequence alignment of the intergenic spacer (IS) region immediately preceding the start codon of the CP gene showed that the region was highly variable, rich in the bases A and T, and that its size varies from 48 nts to 63 nts (Fig. S1 in File S2). In the IS-based phylogenetic tree, most isolates occupied similar positions to those they occupied in the CP-based phylogenetic tree, indicating that the IS sequence diversity of PBNSPaV isolates may reflect the phylogenetic relationships between viral variants.

### Genome sequences of three PBNSPaV isolates

The complete genome sequences of Pch-WH-1, Plm-WH-3, and Pch-GS-3 consisted of 14,208, 14,211, and 14,211 nts, respectively (Table 2). The sequences have been deposited in the GenBank database with accession numbers KJ792852, KJ7928523, and KJ792854. The Pch-WH-1 and Plm-WH-3 isolates had highly similar genome sequences, with 94.8% identity at the nt level, and over 98% identity at the aa level, except for the polymerase encoded by ORF1. The isolate Pch-GS-3 shared only 88.9–89.7% nt identity with isolates Plm-WH-3 and Pch-WH-1. The genome-wide nt sequence identities between these three isolates and the type PBNSPaV isolate PL186 (EF546442) were 90.0% (Pch-GS-3) and ∼96% (Pch-WH-1 and Plm-WH-2), and these three isolates showed low identity (70.2–71.2%) to an isolate Nanjing (KC590347). The genome structure of these three isolates was identical to that of the type PBNSPaV isolate (EF546442), and consisted of seven ORFs. Each ORF was the same size as the corresponding ORF in EF546442, except ORF1b, which was three nts smaller, and the gene encoding the minor coat protein (CPm), which was 16 nts longer than the corresponding genes of isolates Pair-2 and Nanjing. In general, the 3′-UTRs were highly conserved and were 99.0% identical to those of EF546442. ORF1 showed the highest variability, and the three isolates showed 88.2–95.8% nt and 86.5–96.4% aa sequence identities overall to EF546442, and only 66.3–67.6% nt and 67.3–70.6% aa sequence identities to the isolate from Nanjing. The CP and p6 genes appeared to be the most highly conserved proteins, sharing over 93% and 91.2% aa identities among these three isolates and the four reference PBNSPaV isolates. The Pch-GS-3 isolate was the most divergent of the three isolates sequenced here. The 5′-UTR of the Pch-GS-3 isolate was only 87.8% identical to that of EF546442, and its seven ORFs showed overall 88.2–94.3% nt identities to those of EF546442.

**Table 2:** Sequence comparison of the complete genome and different genomic regions between the typical isolate PL186 and three PBNSPaV isolates determined in this study and three isolated referred from GenBank.

isolate	Genome		5′UTR		Pol			RdRp			P6			hHSP70			P61			CP			CPm			3′UTR	
	nt	nt%	nt	nt%	nt	nt%	aa%	nt	nt%	aa%	nt	nt%	aa%	nt	nt%	aa%	nt	nt%	aa%	nt	nt%	aa%	nt	nt%	aa%	nt	nt%
PL186-EF546442	14214		301		7032			1572			171			1587			1638			975			669			231	
Pch-WH-1	14208	96.4	301	97.7	7032	95.8	96.4	1572	97.3	98.9	171	98.3	98.2	1587	97.3	98.1	1638	96.3	98.2	975	96.4	97.8	669	96.9	96.9	231	99.6
Plm-WH-3	14211	95.1	301	96.7	7032	93.7	91.8	1572	96.3	97.1	171	97.7	98.2	1587	96.2	98.1	1638	95.9	97.3	975	96.8	97.5	669	97.8	96.9	231	99.6
Pch-GS-3	14211	90.0	301	88.7	7032	88.2	86.5	1572	92.6	95.2	171	94.3	93.0	1587	91.3	90.9	1638	90.3	89.6	975	92.1	96.0	669	94.3	93.7	231	99.6
TaTao25-KC590344	14211	91.8	301	88.0	7032	90.4	91.5	1572	92.4	96.9	171	97.1	98.2	1587	93.7	96.6	1638	93.3	95.4	975	92.5	96.0	654	95.0	94.5	234	99.1
Pair-2-KC590345	14219	83.1	302	76.7	7035	80.7	84.4	1575	85.4	94.1	171	87.7	98.2	1587	85.1	94.5	1638	83.9	90.8	975	85.9	94.2	654	90.8	89.0	234	98.7
Nanjing-KC590347	14234	71.2	301	61.9	7035	67.6	70.3	1575	74.4	83.8	171	73.7	93.0	1587	75.2	87.1	1638	71.7	83.7	975	76.2	91.1	654	78.3	77.1	234	94.8

**Figure 3:**
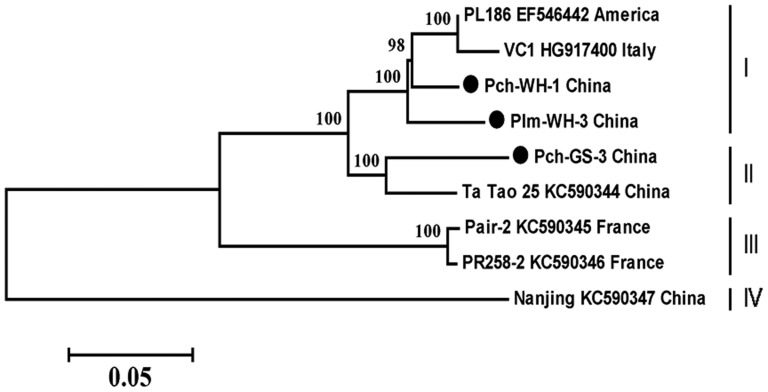
Phylogenetic tree of the complete genome sequences of the three Chinese PBNSPaV isolates identified in this study and six isolates from GenBank. The tree was constructed using neighbor-joining analysis. The reference isolates are identified using their GenBank accession numbers. Bootstrap analysis with 1000 replicates was performed. Only >60% bootstrap values are shown.

Phylogenetic analysis of the complete genomic sequences of the three isolates examined in the present study and the five isolates available in GenBank revealed four groups with 100% bootstrap support values (Fig. 3). Pch-WH-1 and Plm-WH-3 clustered with the type isolate, forming a group I, and Pch-GS-3 clustered with a Ta Tao 25 isolate from a Chinese Ta Tao sample in group II, two isolates (Pair-2 and PR258-2) from France form a group III, and one isolate from Nanjing, China [16], forming a group IV.

### Genetic parameters confirms the genetic diversity of PBNSPaV population

The genetic distance within and between HSP70h groups was calculated using MEGA 5 [24]. The within-group genetic distance was highest in group I (0.052) and lowest in group III (0.009). The between-group genetic distance ranged from 0.167 to 0.342 (Table 3). Group IV, consisting of peach isolates from Gansu province, was found to have the greatest genetic distance (0.332–0.342) from the other three groups. The Nanjing isolate, which was not included in any of the groups, showed the greatest genetic distance (0.335) from group I and the lowest distance (0.211) from group VI. These data strongly supported the topology of the tree inferred from the HSP70h sequences, with four major groups.

**Table 3:** Genetic distances between and within variant groups of PBNSPaV partial HSP70 gene and complete CP gene.

Group^a^	HSP70h				CP			
	Between variant groups			Within variant groups	Between variant groups			Within variant groups
Gp-I				0.05244				0.05427
Gp-II	0.16650			0.00915	0.16757			–
Gp-III	0.18705	0.17264		0.01872	0.16369	0.17544		0.01266
Gp- IV	0.33714	0.34149	0.33186	0.04051	–	–	–	–

a The isolate Nanjing (KC590347) is not included in this analysis.

HSP70h and CP differed in diversity, polymorphic sites, and number of haplotypes (Table 4). An analysis of all available data showed that 47.8% of the nucleotides in HSP70h were polymorphic and 39.1% were parsimony-informative, with a diversity statistic of 0.10521. In the CP gene, 44.8% of the nucleotides were polymorphic and 28.7% were parsimony-informative, with a diversity statistic of 0.06582. These results confirmed that HSP70h was more variable than CP.

**Table 4:** Phylogenetically informative statistics for HSP70h and CP genes.

Statistic	HSP70	CP
Number of polymorphic sites	271	429
Single variants	56	154
Parsimony informative	215	275
Invariant	279	528
Number of haplotypes	93	65
Diversity (θ)c	0.10232	0.06582

The population genetic parameters of the partial HSP70 (590 nt) and CP gene sequences obtained in this study and those available in GenBank were calculated using Dnasp5 (Table 5). The overall nucleotide diversity (π) values for HSP70h and CP were found to be less than 1 and the haplotype diversity (Hd) values were found to be 0.996 and 1.000, indicating low genetic diversity. Negative values were obtained for the Fu and Li’s D statistical tests, suggesting that the viral population may be in expansion; however, these values were not statistically significant. The ratios (dN/dS) of nucleotide substitutions at non-synonymous (dN) to synonymous positions (dS) for HSP70h and CP were 0.083 and 0.099, much less than 1, suggesting that the two proteins have been under strong purifying selection.

**Table 5:** Population genetic parameters and neutrality tests calculated for the HSP70 and CP gene of PBNSPaV on the basis of variant groups and geographical origin.

	*n*	Hd	*π*	Fu & Li’s *D*	Fu & Li’s *F*	dNs	dS	dNs/dS
HSP70	113	0.996	0.10232	−0.95705	−1.09359	0.02739	0.32974	0.083
CP	67	1.000	0.06582	−2.26314	−2.43963*	0.02107	0.21313	0.099

*Statistical significance: P<0.05.

### Selective pressure analyses identified positively selected codons in the PBNSPaV CP gene

Furthermore, the strength of the selective forces acting on codons of the two genes were analyzed using three complementary methods, namely, SLAC, FEL, and IFEL [25]. The results showed that the distribution of codon positions under purifying, neutral, and positive selection differed between the two genes, indicating that the two genes had been exposed to different selective pressures (Table 6). The CP gene contained more neutral codons than HSP70h. Most of the codons in the CP gene were found to be under purifying selection. Five codon positions (3, 8, 44, 57, and 88) of the CP gene were detected to be under positive selection; three of these (3, 8, and 88) were identified by all three methods. However, no codons in the HSP70h gene were found to be under positive selection.

**Table 6:** Estimates of selection pressures acting on the HSP70h and coat protein (CP) genes of global PBNSPaV isolates.

Coding region^a^	Selection pressure^b^								
	Normalized d*N*-d*S* c		Detection methods	Positive		Negative		Neutral	
	Log (L)	Mean		No.^d^	%	No.	%	No.	%
HSP70h	−4306.36	0.0811	SLAC	0	0	99	54.1	84	45.9
			FEL	0	0	136	74.3	47	25.7
			IFEL	0	0	133	72.7	50	27.3
CP	−6404.63	0.1199	SLAC	3 (3, 8, 88)	0.9	133	41.7	183	57.4
			FEL	5 (3, 8, 44, 57, 88)	1.6	201	63.6	113	35.4
			IFEL	4 (3, 8, 44, 88)	1.3	179	56.1	136	42.6

a Data sets are obtained by the analysis of 183 codons for HSP70h region and 319 codons for CP region, respectively.

b Positively or negatively selected sites are identified by four selection pressure detection methods: single-likelihood ancestor counting (SLAC), fixed-effects likelihood (FEL), internal fixed-effects likelihood (IFEL), and random-effects likelihood (REL).

c Normalized values of the ratio of nonsynonymous substitutions per nonsynonymous site (dN) to synonymous substitutions per synonymous site (dS) (dN–dS). Normalized dN–dS values <1 indicates negative or purifying, normalized dN–dS values = 1 suggests neutral selection, and normalized dN-dS values>1 indicates positive selection.

d The positively selected amino acid sites are indicated in brackets.

### The HSP70h gene of PBNSPaV exhibits significant recombination

One HSP70h variant sequence of a previously reported peach isolate, Pch2 (JF810178), showed significant evidence of recombination based on analysis using RDP3 (MaxChi×1.356 10^−03^, SiScan 7.128×10^−06^, LARD 1.313×10^−01^, 3Seq 3.023×10^−05^, Chimaera 8.270×10^−04^). Recco analysis showed that the recombination junction was between nt 313–331 (Fig. S2 in File S2), with Pch3 (JF810179) and Pch1 (JF810177) identified as the major and minor parents, sharing 98.73% and 93.82% nt, and 98.91% and 97.81% aa similarities, respectively. Moreover, two other HSP70h variant sequences, Apr-YT-1-3 from apricot and Pch2-3 from peach, showed potential evidence of recombination based on Recco analysis (Fig. S2 in File S2). Of those recombinants, variant Pch2-3 and Pch2 (JF810178) were obtained from the same peach plant. However, the variant Pch2-3 showed evidence of a different recombination junction at nts 76–94, with Pch2-1 and Pch6-3 as its parents. The recombination junction of Apr-YT-1-3 was found to be between nts 233 and 245; Ch-YT-2-1 was identified as one of its parents, and the other parent was unknown.

## Discussion

Of the 256 *Prunus* samples examined in the present study, 56 were found to be infected by PBNSPaV, representing an incidence of 20.3% in stone fruit trees, similar to results obtained for samples collected in Italy [26], but much higher than the incidence identified in other studies [16]. The wide species of stone fruit hosts identified and the high incidence of PBNSPaV suggest that the virus may be efficiently transmitted in nature, similar to other closteroviruses.

To date, only a few partial sequences of the HSP70h gene of PBNSPaV are available, and no studies have been conducted on the phylogenetic characteristics of the CP gene, with the exception of the six fully sequenced isolates. In this study, sequence analyze of a 590-bp fragment of HSP70h from 31 isolates and the complete CP gene from 38 isolates shows that the Chinese PBNSPaV population consists of highly divergent isolates, which can be divided into four distinct HSP70h clusters. Two HSP70h groups, II and IV, consist solely of Chinese PBNSPaV isolates sequenced in the present study and are likely to represent two novel phylo-groups. Especially, group IV consisting of molecular variants of four peach isolates from Gansu province were the most divergent from the other groups, with genetic distances of 0.332–0.342, and a distance of 0.207 from the Nanjing isolate. Unfortunately, CP gene sequences of the isolates in HSP70h group IV could not be obtained, although several primer pairs were designed in attempts to amplify their CP gene. The failure of primers that successfully amplified most isolates to amplify CP gene of isolates Pch-GS-1, 2, 3 and 4 suggests a high level of sequence divergence in the genome of these isolates as compared to the other isolates. Marais *et* *al.* experienced a similar problem [16]. The similar topologies of the phylogenetic trees constructed using all available CP and HSP70h sequences from PBNSPaV isolates indicate a co-evolutionary tendency between the two genes [27]. However, phylogenetic analyses did not show a clear relationship between genetic variability of PBNSPaV isolates and geographical or host origin excerpt that a few sequences (Pch-GS-1, 2, 3 and 4) from peach grown in Gansu formed a separated group. Furthermore, to the best of our knowledge, our study is the first to show the occurrence of mixed infections of highly divergent variants of PBNSPaV in individual host plant. The Pch-GS-3 and Pch-XN-3 isolates contained HSP70h variants in groups I and IV and CP variants in groups I and II, respectively. The presence of divergent variants in a single host plant may be a result of different infection events during horticultural operations or of vector (if it presents) transmission. Mixed infections with divergent variants could increase viral genotypic complexity, with implications for phylogenetic analysis and the evolutionary history of the virus.

Analysis of the complete genome sequences of three Chinese PBNSPaV isolates determined in this study, combined with two Chinese PBNSPaV isolates (Ta Tao 25 and Nanjing) sequenced by other researchers [16] also showed the genetic complexity of the Chinese PBNSPaV population. The genomic sequences of Pch-WH-1 from a peach and Plm-WH-3 from a plum were closely related to the type isolate PL186, sharing 96% overall nucleotide identity. However, Pch-GS-3 was found to be closely related to Ta Tao 25 (92.7% overall nucleotide identity), but less closely related to Plm-WH-3 and Pch-WH-1. Although Pch-WH-1 and Plm-WH-3 recovered from the same region (Wuhan) share high similarity of 94.8%, we cannot postulate that the sequence similarity is related to their same geographic origin. This similarity could be related to origin of their foremost isolate, from which variants were separated during dissemination. In addition, although it has been observed that the infection of PBNSPaV can induce severe symptoms or be latent, more extensive studies will be done to reveal the relationships between host phenotypes and viral genotypes. Moreover, the clustering pattern of the complete genome sequences was substantially concordant with the topology of the HSP70h-derived trees, indicating that the evolutionary relationship of global PBNSPaV isolates can be reliably inferred using HSP70h sequences.

Recombination is a powerful driving force for generating new variants in RNA viruses [28]–[30]. Recombination events have been reported to be evolutionarily important in shaping the genomes of some viruses in the family *Closteroviridae* [27]. Multiple recombination events have been identified between sequence variants of the *Citrus tristeza virus* [31]–[33]. As more sequences of PBNSPaV variants become available, we may be able to identify more potential recombination events between different variants. The presence of divergent PBNSPaV variants and co-infection with different variants enable the occurrence of recombination events. For the first time, we report evidence of significant recombination in the HSP70h gene of PBNSPaV variant Pch2. Another HSP70h variant, Pch2-3, also from the Pch2 isolate, and two other variants, Apr-YT-3-1 and Pch-XN-4-6, also showed clear crossover sites at regions having high sequence similarity with their parental variants [34], although these were not detected by the programs implemented in RDP3. However, no recombination events were detected in the CP gene. The results indicate that the HSP70h gene could be a hotspot of recombination in the PBNSPaV genome; our results contrast with those from GLRaV-3, which has been shown to contain recombination hotspots in its CP gene [35], although both viruses are in the same genus.

The HSP70h genes of plant viruses in the family *Closteroviridae* have multiple biological functions and are used for phylogenetic classification of viruses in this family [1]. Most plant closteroviruses are insect-vector transmissible [27]. Thus, their capsid proteins play important roles in their ability to survive in both plant and insect environments. To date, no insect vector has been identified for PBNSPaV. Although mealybugs were frequently observed on PBNSPaV-infected fruit trees during this investigation, no insects were found to be PBNSPaV-positive in RT-PCR tests (data not shown). Some studies have indicated that vector-borne plant viruses are subjected to greater purifying selection on their capsid proteins than non-vectored viruses [36]. The population genetic parameters of both the HSP70h and CP genes suggest the possibility of a PBNSPaV population in expansion. Given the low dN/dS ratios found for HSP70h and CP, combined with the large portion of negatively selected sites identified in both genes, it appears that the virus may have undergone purifying selection, which is the primary evolutionary force acting on many plant viruses [37]. In addition, although HSP70h is more variable than CP as it is showed in Table 4, the higher dNs/dS value and the presence of positively selected sites in the CP gene indicate that the CP gene is under stronger selection pressure than that acting on the HSP70 gene. In accordance with the expectation that most mutations are deleterious or lethal to plant RNA viruses due to the compactness of viral genomes [38], the large portion of negatively selected sites existing in those two gene. The presence of a small number of positively selected sites in the CP gene might be necessary for adaption to different biological conditions. Since the successful infection of a virus in hosts depends on multiplex interactions between the virus and its host, beneficial mutations (positively selected sites) may affect interactions with host receptors and other host-specific molecules [39]. Extensive investigations will be necessary to generate detailed information about the viral population structure and the biological and epidemiological implications of its genetic diversity and possible transmission vectors.

Because the genetic diversity within global PBNSPaV populations has not been studied extensively, the results of this study provide important information on the genetic diversity of the Chinese PBNSPaV population and will advance the improved understanding of the epidemiology of the virus on a global scale and give insights into viral disease management [40]. From a practical perspective, the genetic diversity data generated in this study show that the HSP70h-specific primer pair is more robust and better, and that it is able to detect more PBNSPaV isolates than the primers used to amplify the CP gene. Considering the economic impact of the virus on many stone fruits [41], it is necessary and urgent to establish an efficient scheme for reducing the global transmission of the virus via propagation materials.

## Materials and Methods

### Sample collection

In total, 256 samples from 7 species of *Prunus*, including 98 from peach (*P. persica*), 49 from plum (*P. domestica*), 50 from sweet cherry (*P. avium*), 10 from flowering cherry (*P. serrulata*), 24 from flowering peach (*P. persica*), 18 from apricot (*P. armeniaca*), and 7 from nectarine (*P*. *persica* var. *nucipersica*), were collected in China between 2009 and 2013. All sample collections were conducted with approval from local institutes, and no specific permissions were required for these locations/activities. The study did not involve endangered or protected species. Symptoms of sampled trees consisting of stem pitting on tree trunks with thick corky bark, and dark-colored gummosis or spongy texture and severe cracks in the bark were observed on the trunks of some peach and plum trees. Virus-free seedlings of peach GF305 were used as negative controls in all tests for the detection of the virus.

The complete genome sequences of three PBNSPaV isolates, namely, Pch-WH-1, Plm-WH-3, and Pch-GS-3, were determined. Isolates Pch-WH-1 and Plm-WH-3 were collected from a peach tree and a plum tree grown in Wuhan city in central China, and the isolate Pch-GS-3 was taken from a peach tree grown in the Gansu province in western China. Based on our primary results of HSP70h sequencing, the three isolates distributed into three phylogenetic clades or sub-clades. Both peach tree infected by Pch-WH-1 and plum tree infected by Plm-WH-3 showed visible symptoms (Table 1) and represent two important host species of the virus.

### RT-PCR and Sequencing of HSP70h and CP genes

Total RNA was extracted from leaves using a CTAB protocol, as described by Li *et* *al.* [42]. The presence of PBNSPaV was tested by reverse transcription (RT)-PCR using the primer set PBN-195-F/PBN-195-R (5′-CTGGTCTTCCTGCTACTCCTT-3′/5′-CGCTCTGAGATTGTGGGCTT -3′), designed for the detection of the coat protein (CP) gene of the virus [17].

The primer pairs PBN-HSP-F/PBN-HSP-R (5′-GGAATTGACTTCGGTACAAC-3′/5′-TTCGGTGGTGGTACTTTCGA-3′) and PBN-CP-F/PBN-CP-R (5′-TCTTGTTGGATCGGGGAATA-3′/5′-CATCTTCCACCGGACTGATT-3′), designed based on the corresponding sequences of the American PBNSPaV isolate PL186, were used for the amplification of partial HSP70h gene sequences and complete CP gene sequences, respectively. Reverse transcription was performed at 37°C for 1.5 h using 3 µL of total RNA and 1 µL of random primer in a 20-µL reaction volume with Maloney murine leukemia virus (M-MLV) reverse transcriptase (Promega, Madison, WI, USA), according to the manufacturer′s protocols. PCR was performed in a 25-µL volume of reaction mixture containing 2.5 µL of 10×PCR buffer, 0.5 mM dNTPs, 0.5 mM of each primer, one unit of *Taq* DNA polymerase (TaKaRa, Dalian, China), and 3 µL cDNA. PCR was performed using an iCycler Thermocycler (Bio-Rad, Hercules, CA). The PCR profile employed for all primer sets consisted of an initial denaturation at 95°C for 30 s followed by 35 cycles of 95°C for 3 min, 52°C for 30 s, 72°C for 1 min, and a final extension for 10 min at 72°C. The PCR products were separated by electrophoresis on a 1.2% agarose gel, stained with ethidium bromide, and visualized under UV light.

PCR products were gel purified and ligated into the pMD18-T vector (Takara, Dalian, China), following the manufacturer’s instructions. The recombinant plasmids were identified after transformation into *Escherichia coli* DH5α. In order to obtain a view of molecular composition intra each isolate, at least three positive clones of each product were sequenced at Shanghai Sangon Biological Engineering & Technology and Service Co. Ltd, Shanghai, China.

### Determination of complete genome sequences

For the amplification of PBNSPaV genomes, primer sets (Table S2 in File S1) were designed based on the CP and HSP70h gene sequences obtained, and then the amplifications were extended toward the 3′- and 5′-ends using primer sets designed based on the sequences obtained here and the genome sequence (EF546442) of the first PBNSPaV isolate PL186, available in GenBank. 5′- and 3′-RACE reactions were attempted using an Invitrogen GeneRacer Kit (Invitrogen, USA), according to the manufacturer’s instructions. PCR solutions and conditions were similar to those mentioned above, except that 2 mM of each dNTP was used in a 25-µL reaction volume, with annealing for 45 s at 50–54°C (depending on the primer set used in each reaction), with extension for 1–3 min (depending on the size of the PCR product) at 72°C. All products were cloned and sequenced as mentioned above. To overcame inconvenient caused by intra-isolate sequence diversity and avoid mistakes in sequence assembling, the adjacent amplicons were overlapped for >100 bp, and at least three clones of each PCR product were sequenced. The obtained sequences were assembled into a contiguous sequence at a standard of over 99.9 % similarities at each of overlapped regions.

### Phylogenetic and recombination analyses

The sequence alignment (produced using Clustal W) was imported into the program MEGA5, and the phylogenetic tree was constructed using the neighbor-joining method with 1,000 bootstrap replicates [24], [43]. The number of polymorphic sites, single variants, parsimony-informative sites, invariant sites, haplotypes, and diversity (θ) were determined using Dnasp5 software package. To obtain an overview of the Chinese PBNSPaV population, the eighteen HSP70h sequences reported by Cui *et* *al* [17], the complete genome sequences of the five PBNSPaV isolates available in GenBank, and the two HSP70h sequences available in GenBank were included in the corresponding sequence analyses. The sequence sources and GenBank accession numbers of the PBNSPaV isolates used in the sequence analysis are listed in Table S1 in File S1.

Finally, the sequence alignments for the HSP70h and CP genes were scanned using seven programs for detection of recombination, implemented in the software RDP v.3.27 [44], for evidence of recombination and to determine putative recombination events, potential recombinants, and their parental sequences. Only recombination events detected by at least four methods were included in further analyses. Meanwhile, recombination profiles were constructed by using the software Recco [45].

## Supporting Information

File S1 **Supporting tables. Table S1, The sources of HSP70h gene and complete genome of PBNSPaV isolates referred from GenBank. Table S2, Primers used for the amplification of the genomes of PBNSPaV isolates Pch-WH-1, Plm-WH-3 and Pch-GS-3.**
(DOCX)

File S2 **Supporting figures. Figure S1, Nucleotide sequence alignments (A) and phylogenetic analysis (B) of the intergenic spacer (IS) region.** The tree was constructed using the maximum likelihood method. **Figure S2, The Recco output for molecular variants Pch2, Pch2-3, Apr-YT-3-1, and Pch-XN-4-6, based on their HSP70h sequences.** The possible crossover sequences for each variant are marked by two arrows. The possible recombinant sequences are shown in white, and sequences highly similar to recombinant sequences are marked in red.
(PPTX)
