# Supplementary material for: Genetic Diversity and Molecular Evolution of Plum bark necrosis stem pitting-associated virus from China
Source: PLoS One. 2014 Aug 21;9(8):e105443. doi: 10.1371/journal.pone.0105443 (PMC4140750; doi:10.1371/journal.pone.0105443)
Supplement: File S1 — Supporting tables. Table S1, The sources of HSP70h gene and complete genome of PBNSPaV isolates referred from GenBank. Table S2, Primers used for the amplification of the genomes of PBNSPaV isolates Pch-WH-1, Plm-WH-3 and Pch-GS-3. (DOCX) [file pone.0105443.s001.docx]

**Table S1** The sources of HSP70h gene and complete genome of PBNSPaV isolates referred from GenBank

| Gene | Isolate | Country | Host | Accession numbers | References |
| --- | --- | --- | --- | --- | --- |
| HSP70h | Unknown | America | plum | AF195501 | Unpublished |
|  | Unknown | Italy | apricot | AJ305307 | Unpublished |
|  | Pch1 | China | peach | JF810177 | Cui *et al.*, 2011 |
|  | Pch2 | China | peach | JF810178 |  |
|  | Pch3 | China | peach | JF810179 |  |
|  | Pch4 | China | peach | JF810180 |  |
|  | Pch5 | China | peach | JF810181 |  |
|  | Pch6 | China | peach | JF810182 |  |
|  | Pch7 | China | peach | JF810183 |  |
|  | Nec18 | China | nectarine | JF810194 |  |
|  | Plm12 | China | plum | JF810188 |  |
|  | Plm13 | China | plum | JF810189 |  |
|  | Fplm14 | China | flowering plum | JF810190 |  |
|  | Fchr16 | China | flowering cherry | JF810192 |  |
|  | Fchr17 | China | flowering cherry | JF810193 |  |
|  | Chr15 | China | cherry | JF810191 |  |
|  | Fpch8 | China | peach | JF810184 |  |
|  | Fpch9 | China | peach | JF810183 |  |
|  | Fpch10 | China | peach | JF810186 |  |
|  | Fpch11 | China | peach | JF810187 |  |
| genome | PL186 | America | plum | EF546442 | Al Rwahnih *et al.*, 2007 |
|  | TaTao25 | China | peach | KC590344 | Armelle *et al.*, 2014 |
|  | Pair-2 | France | plum | KC590345 |  |
|  | PR258-2 | France | plum | KC590346 |  |
|  | Nanjing | China | peach | KC590347 |  |
|  | VC1 | Italy | apricot | HG917400 | Unpublished |

**Table S2** Primers used for the amplification of the genomes of PBNSPaV isolates Pch-WH-1, Plm-WH-3 and Pch-GS-3

| **Fragments** | **Primer** | **Primer sequence (5′→3′)** | **Product sizes (bp)** | **Position** (nt)^a^ | **Target isolates** |
| --- | --- | --- | --- | --- | --- |
| F1 (5’end) | 5’RACE Inner | CGCGGATCCACAGCCTACTGATGATCAGTCGATG | 796 | 738-757 | Pch-WH-1, Plm-WH-3, Pch-GS-3 |
|  | PBN-5-R1 | TGTTCCACATCCGGTAACTG |  |  |  |
|  | 5’RACE Out | CATGGCTACATGCTGACAGCCTA |  | 843-862 |  |
|  | PBN-5-R | TGAGGCTGTAGCGAAACTGA |  |  |  |
| F2 | PBN-C-F1 | GCCGTTTACCAGCAGTTTCT | 2438 | 689-708 | Pch-WH-1, Plm-WH-3 |
|  | PBN-C-R1 | TTGTTCTTATCCTGGGGCTG |  | 3117-3126 |  |
|  | PBN-C-F1 | GCCGTTTACCAGCAGTTTCT | 2471 | 689-708 | Pch-GS-3 |
|  | PBN-F2-R1 | TCACGGTTTGTAGGGAAGGT |  | 3140-3159 |  |
| F3 | PBN-B-F1 | AAACCCTGTGGTGAGTGTGA | 2105 | 2956-2975 | Pch-WH-1, Plm-WH-3, Pch-GS-3 |
|  | PBN-B-R1 | TGGTGATAAAGCTTACCTCGC |  | 5040-5060 |  |
| F4 | PBN-A-F1 | GAGTAATGGTTGCGGGTTGT | 2894 | 4718-4737 | Pch-WH-1, Plm-WH-3 |
|  | PBN-A-R2 | AAATTCTCTCGTCGCTTGGA |  | 7592-7611 |  |
|  | PBN-A-F3 | TATACCCTTAGCCGGGTTGG | 2754 | 4806-4825 | Pch-GS-3 |
|  | PBN-A-R3 | AAATTCTCTCGTCGCTTGGA |  | 7540-7559 |  |
| F5 | PBN-R-F | CATAGGCTTGTGGGATTAAC | 2026 | 7180-7199 | Pch-WH-1, Plm-WH-3 |
|  | PBN-R-R1 | GATGGGACTTTTTGTATCGG |  | 9186-9205 |  |
|  | PBN-F5-F1 | ACAGGGGTAGACAGGCTGAA | 2233 | 7136-7154 | Pch-GS-3 |
|  | PBN-F5-R1 | TTGGACCTGTGTCCGGTAAT |  | 9349-9368 |  |
| F6 | PBN-HSP-P1 | GGAATTGACTTCGGTACAAC | 1712 | 9069-9088 | Pch-WH-1, Plm-WH-3, Pch-GS-3 |
|  | PBN-HSP-R1 | GGTGAAAGGGAACATACGGA |  | 10761-10780 |  |
| F7 | PBN-HSP-F1 | TTCCAAATCACCGAGTGACA | 790 | 10595-10614 | Pch-WH-1, Plm-WH-3 |
|  | PBN-P61-R1 | ACCAGTGACACTTCATTAAG |  | 11365-11384 |  |
|  | PBN-HSP-F1 | TTCCAAATCACCGAGTGACA | 754 | 10595-10614 | Pch-GS-3 |
|  | PBN-F7-R | GCTTCCCAAGTTGCAAGTTT |  | 11329-11348 |  |
| F8 | PBN-P61-F | CTTTACCAGTTTCTTCACGA | 2134 | 11180-11199 | Pch-WH-1, Plm-WH-3, Pch-GS-3 |
|  | PBN-CP-R | AATCAGTCCGGTGGAAGATG |  | 13294-13313 |  |
| F9 (3’end) | PBN-3-F1 | CGAAACCGGATATGGAGCTA | 1031 | 12926-12945 | Pch-WH-1, Plm-WH-3, Pch-GS-3 |
|  | 3’RACE Out | TACCGTCGTTCCACTAGTGATTT |  |  |  |
|  | PBN-3-F | GCGAGTCAGTTGGGTCGTAT |  | 13183-13222 |  |
|  | 3’RACE Inner | CGCGGATCCTCCACTAGTGATTTCACTATAGG |  |  |  |

^a^ nt positions are determined from the genome sequence of the isolate PL186 of PBNSPaV (GenBank accession No. EF546442).
