# Supplementary figures and images for: Genetic Diversity and Molecular Evolution of Plum bark necrosis stem pitting-associated virus from China
Source: PLoS One. 2014 Aug 21;9(8):e105443. doi: 10.1371/journal.pone.0105443 (PMC4140750; doi:10.1371/journal.pone.0105443)

## Slide 1
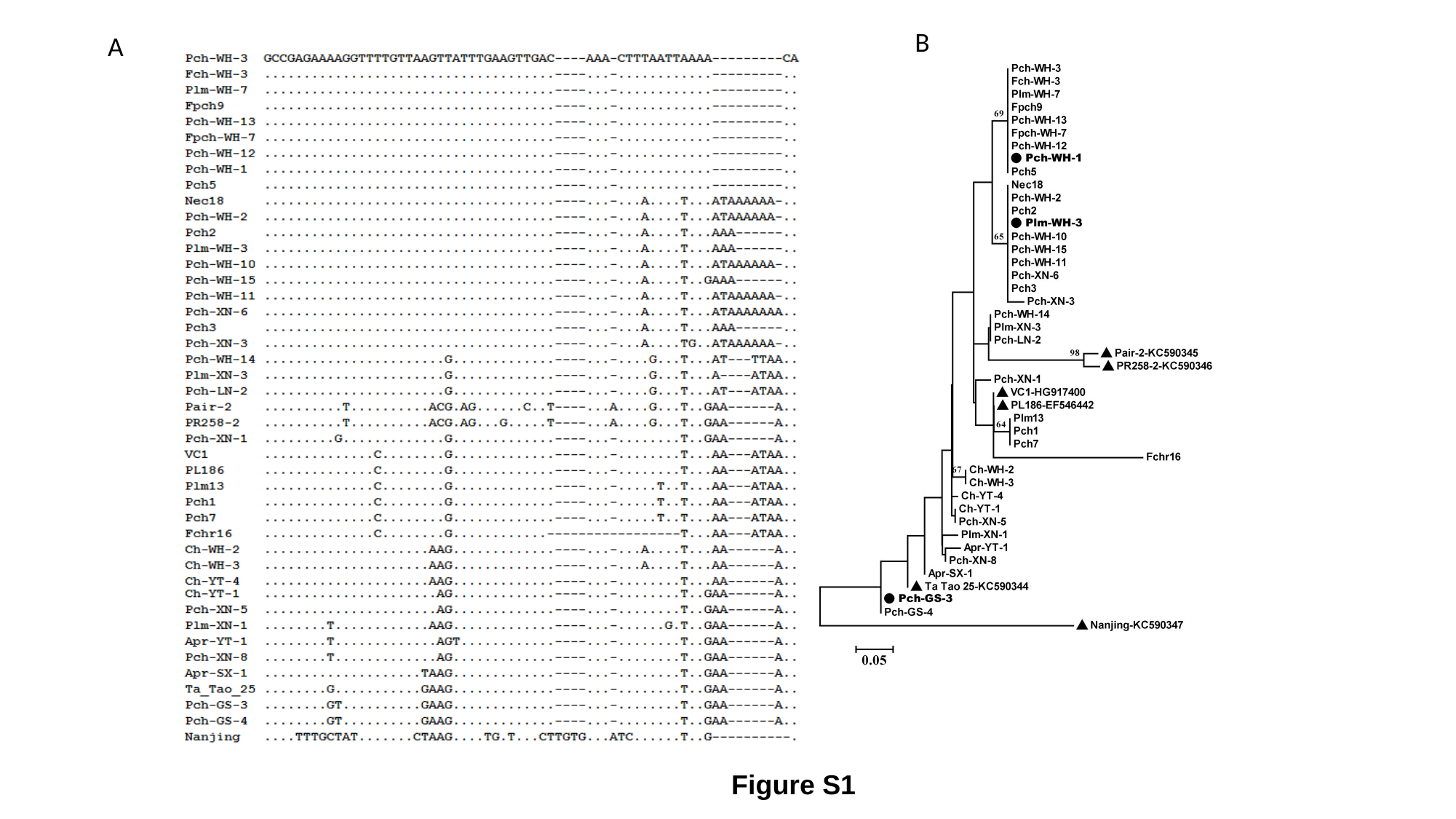

B
A
Figure S1

## Slide 2
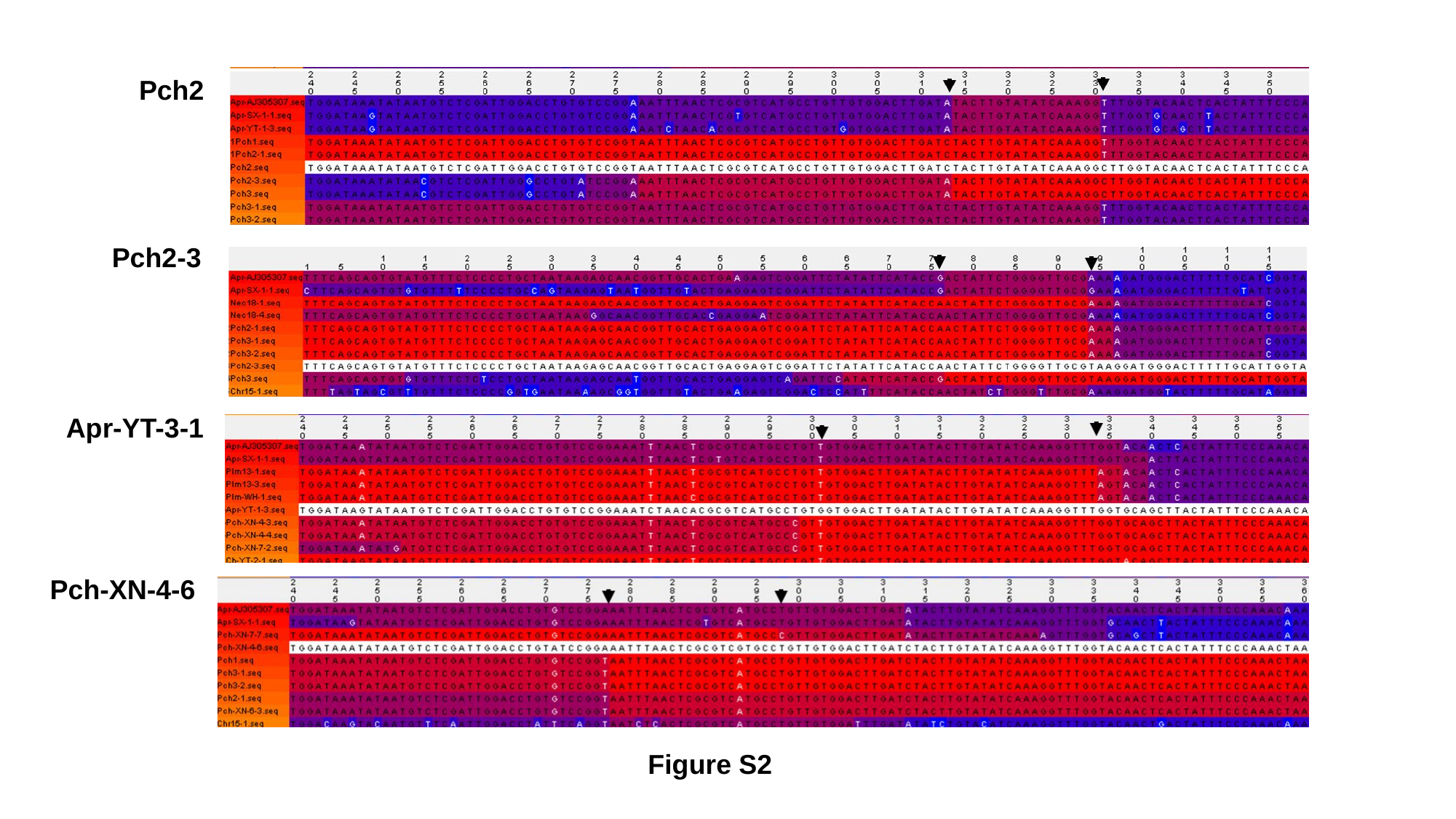

Pch2
Pch2-3
Apr-YT-3-1
Pch-XN-4-6
Figure S2

Supplement: File S2 — Supporting figures. Figure S1, Nucleotide sequence alignments (A) and phylogenetic analysis (B) of the intergenic spacer (IS) region. The tree was constructed using the maximum likelihood method. Figure S2, The Recco output for molecular variants Pch2, Pch2-3, Apr-YT-3-1, and Pch-XN-4-6, based on their HSP70h sequences. The possible crossover sequences for each variant are marked by two arrows. The possible recombinant sequences are shown in white, and sequences highly similar to recombinant sequences are marked in red. (PPTX) [file pone.0105443.s002.pptx]
